# Inverse Association Between the Mediterranean Diet and COVID-19 Risk in Lebanon: A Case-Control Study

**DOI:** 10.3389/fnut.2021.707359

**Published:** 2021-07-30

**Authors:** Christine N. El Khoury, Sofi G. Julien

**Affiliations:** Department of Nutrition and Food Sciences, Faculty of Arts and Sciences, Holy Spirit University of Kaslik, Jounieh, Lebanon

**Keywords:** COVID-19, Mediterranean diet, Lebanon, immunity, dietary patterns, nutrition, western diet

## Abstract

**Background:** Since 2019, the world is confronting the COVID-19 public health crisis that deeply impacted all aspects of life, from the health sector to economy. Despite the advancement of research targeting pandemic containment measures, more strategies are still needed to alleviate the burden caused by this novel disease. In particular, optimal nutrition was proposed as a possible mitigating factor in the context of COVID-19. Indeed, the light is shed on balanced diets, such as the Mediterranean diet, which present the finest nutritional quality to support the immune system and other physiologic functions. In contrast, less varied diets that lack the needed nutrients and favor inflammation have been correlated with adverse health effects, including a hindered immune response, such as the western diet.

**Methods:** This observational case control study aimed at exploring the possible associations between the different dietary patterns present among a sample of the Lebanese population and the COVID-19 occurrence and outcomes. An online survey collected information about the sociodemographic characteristics, health status, lifestyle, and dietary habits through the Mediterranean diet questionnaire and a semi-quantitative fod frequency questionnaire, and the COVID-19 infection and its burden. The sample consisted of 399 respondents divided into the case and control groups (37.6 and 62.4%, respectively) on the basis of the presence or absence of a COVID-19 infection history.

**Results:** The participants in the case and control groups had average adherence to the Mediterranean diet and their dietary intake was closer to the western diet. However, the cases had a lower mean of the MedDiet score (*p* = 0.009). Food groups consumption analysis showed that this significant difference within the overall similar dietary patterns was due to a higher consumption of poultry and a trend toward decreased consumption of olive oil and increased read meat and alcohol intake among the cases. Additionally, gender influenced the levels of different foods' consumption. Nonetheless, the dietary intake did not impact the COVID-19 burden.

**Conclusion:** It is recommended to adopt healthy food choices within the different dietary patterns for a better protection against COVID-19. These findings should be validated in larger-scale studies.

## Introduction

The global public health sector has been challenged since the emergence of the novel corona virus disease (COVID-19) in December 2019. The scientific community has made remarkable efforts in order to provide adequate containment strategies for this pandemic. Despite the vaccination campaigns, the hygiene measures and the social restrictions, the overwhelming burden of the COVID-19 pandemic is still a major crisis, and additional management tools are needed (1). For instance, the host's nutritional status has been advocated as a key factor in the context of COVID-19 (2).

Indeed, the pivotal role of nutrition in supporting the immune response has been largely studied (3, 4). Diets that provide optimal nutritional quality are required for immunocompetence, such as the Mediterranean diet (MD), which was associated with a multitude of anti-inflammatory and metabolic benefits (5–7). In contrast, dietary patterns that favor inflammation and lack immune-supporting nutrients, such as the western diet, might hinder the immune response (8). Therefore, based on the available evidence about the complex interactions between diet and immunity, the impact of nutrition on COVID-19 is a topic of interest. In fact, a recent study has found an inverse association between the adherence to the MD and COVID-19 cases and deaths in Spain and twenty-three European countries (9).

On the local level in Lebanon, the population's dietary intake has witnessed a shift toward the western dietary pattern, which is alarming considering the negative effects of such diets on immunity (10). Moreover, Lebanon has been deeply affected by the COVID-19 pandemic in the midst of an unprecedented socioeconomic crisis (11). Thus, all the possible infection- control strategies are needed to flatten the epidemiologic curve and alleviate the pandemic burden.

The crucial need for public health support along with the available evidence about the importance of nutrition in enhancing the immune response, emphasize the need for research projects that tackle the role of the different dietary patterns in the COVID-19 pandemic. To our knowledge, no previous studies have explored this association among the Lebanese population. This study aimed at (i) identifying the dietary patterns of the Lebanese adults, (ii) assessing the dietary differences between COVID-19-infected and non-infected adults, (iii) determining the possible associations between nutrition and the infection occurrence and/or outcomes.

## Materials and Methods

### Study Design and Participants

This observational case control study consisted of an online survey. In order to be eligible for the participation in the study, the respondents had to be Lebanese citizens residing in Lebanon, males or non-pregnant females, aged between 21 and 64 years old. Additionally, the participants should not have had immunocompromising health conditions such as cancer, organ transplant, immune deficiency, human immunodeficiency virus, corticosteroids or other immunosuppressive treatment. This study recruited 693 participants from a simple random sampling and 294 of them did not meet the inclusion criteria and were excluded from the study. A total of 399 participants fulfilled the questionnaire and were segregated into two groups; a case group comprised of 150 participants who previously had a symptomatic COVID-19 infection and who were COVID-19- negative by the time of the questionnaire completion, and a control group that included 249 participants who have never been infected with COVID-19 as summarized in Supplementary Figure 1. The protocol of this study was approved by the ethics committee of the Holy Spirit University of Kaslik. The participants were provided with a consent form to agree upon before filling out the questionnaire.

### Data Collection

An online self-administrated questionnaire was designed in English and Arabic and was created through the QSurvey website. It aimed at collecting information about the participants' health status, lifestyle and dietary habits, sociodemographic characteristics, COVID-19 infection history and symptoms when applicable. The questionnaire consisted of five parts divided as follows: (i) qualification for the participation in the study and COVID-19 infection (Lebanese citizenship, governorate of residence, gender, age, pregnancy (in case of females respondents), immunocompromising health conditions, COVID-19 occurrence, symptoms, hospitalization, and current infection status); (ii) other sociodemographic characteristics (education, employment and marital status); (iii) pre-existing health conditions; (iv) lifestyle habits (smoking and physical activity) and (v) dietary intake. The latter part was divided into two sections. The first section consisted of the Mediterranean diet questionnaire and the second section included a sixteen items semi-quantitative food frequency questionnaire (FFQ).

### Data Processing

Some questions' responses were further categorized into specific classifications. The COVID-19 burden was determined on the basis of the number of symptoms and hospitalization as follows; <5 symptoms: mild burden, between 5 and 10 symptoms: moderate burden, more than 10 symptoms and/or hospitalization: high burden (12). The hospitalized patients were also classified as non-severe cases (no disruption of daily life, hindrance to daily life without oxygen requirement, oxygen treatment via nasal canula, and oxygen mask) or severe cases (mechanical ventilation or multi-organ damage and extracorporeal membrane oxygenation) (13).

Moreover, the physical activity habits of the participants were compared to the guideline of 150 min of moderate physical activity per week. The participants who practiced moderate exercise for at least 150 min per week were considered as meeting this guideline, while those who reported fewer durations of practice were considered as not meeting this guideline (14).

The Mediterranean diet questionnaire assessed the consumption of eleven food groups (non-refined cereals, potatoes, fruits, vegetables, legumes, fish, red meat and products, poultry, full-fat dairy, olive oil, and alcohol). Each food group was attributed a score from 0 to 5, based on the frequency of its consumption. The total “MedDiet score,” which was obtained after adding the scores of all the food groups, ranged from 0 to 55 and higher values represented a higher adherence to the MD (15). The participants were subsequently grouped into three levels of adherence to the MD (between 0 and 22: low adherence, between 23 and 34: average adherence, from 35 to 55: high adherence to the MD) (16).

Regarding the FFQ, the sixteen food groups had four levels of consumption (never, once or twice per week, three to six times per week and daily) (17). The food items were used to identify the respondents' dietary patterns by two *a posteriori* methods: principal component analysis (PCA) and cluster analysis. First, the Kaiser-Meyer-Olkin (KMO) measure of sampling adequacy and the Bartlett test of sphericity were evaluated to justify using the PCA. Varimax rotation allowed the extraction of specific factors after considering the eigenvalues higher than 1 and the scree plot. These factors corresponded to the participants' diets. The rotated factor loading for each item was retained when its value was higher than 0.4. Then, each participant was attributed a score for each extracted factor via the multiple regression method. The score indicated the level of correlation between the individual's dietary intake and the extracted factor/diet (18–20). Second, these scores were used in the K-mean cluster analysis to divide the participants into separate clusters corresponding to their identified dietary pattern (17). Furthermore, the consumption of the different food groups assessed in the two food questionnaires was compared between the participants.

### Statistical Analysis

The statistical tests were performed through the Statistical Product and Service Solutions (SPSS) IBM software (SPSS Inc., Chicago, IL, USA), version 25 for Macintosh. Categorical data were described by percentages while the continuous variable was characterized by mean and standard deviation (SD). For the correlation statistics, Pearson's Chi-square test, Fisher's exact test for variables with more than 20% of the cells having expected counts <5, independent *t*-test and logistic regression were carried out and a P value *p* < 0.05 was considered significant.

## Results

### Sociodemographic Characteristics, Health Status, and Lifestyle Habits

Out of the 693 collected responses, only 399 met the inclusion criteria and were complete questionnaires. The included participants were separated into two groups: the case group that consisted of 150 respondents (37.6%) and the control group that included 249 participants (62.4%). As shown in Table 1, the majority of the cases and controls were females (68 and 71.1%, respectively), residing in the Bekaa governorate (63.3 and 59%, respectively), aged between 21 and 29 years old (50.7 and 49.4%, respectively), single (55.3 and 57.8%, respectively), had a university and/or higher education degree (85.3 and 89.6%, respectively) and were employed (48 and 46.2%, respectively). No statistically significant differences were found between the sociodemographic characteristics of the cases and controls.

**Table 1:** Sociodemographic characteristics, health status and lifestyle habits of the participants.

**Characteristics**	**Total**	**Case group**	**Control group**	***P* value**
	***n* (%)**	***n* (%)**	***n* (%)**	
**Gender**				
Female	279 (69.9)	102 (68)	177 (71.1)	0.515
Male	120 (30.1)	48 (32)	72 (28.9)	
**Governorate of residence**				
*South Lebanon*	3 (0.8)	1 (0.7)	2 (0.8)	0.482
North Lebanon	18 (4.5)	9 (6)	9 (3.6)	
Bekaa	242 (60.7)	95 (63.3)	147 (59)	
Beirut	24 (6)	7 (4.7)	17 (6.8)	
*Nabatieh*	3 (0.8)	0	3 (1.2)	
*Akkar*	3 (0.8)	0	3 (1.2)	
*Baalbek – Hermel*	6 (1.5)	3 (2)	3 (1.2)	
Mount Lebanon	100 (25.1)	35 (23.3)	65 (26.1)	
**Age group in years**				
21–29	199 (49.9)	76 (50.7)	123 (49.4)	0.571
30–39	85 (21.3)	32 (21.3)	53 (21.3)	
40–49	72 (18)	30 (20)	42 (16.9)	
50–59	36 (9)	11 (7.3)	25 (10)	
60–64	7 (1.8)	1 (0.7)	6 (2.4)	
**Educational level**				
*Elementary school*	1 (0.3)	1 (0.7)	0	0.217
*Middle school*	8 (2)	2 (1.3)	6 (2.4)	
High school	39 (9.8)	19 (12.7)	20 (8)	
University and/or higher eduction	351 (88)	128 (85.3)	223 (89.6)	
**Employment status**				
Student	83 (20.8)	22 (14.7)	61 (24.5)	0.106
Not working	58 (14.5)	22 (14.7)	36 (14.5)	
Employed	187 (46.9)	72 (48)	115 (46.2)	
Self-employed	65 (16.3)	31 (20.7)	34 (13.7)	
Retired	6 (1.5)	3 (2)	3 (1.2)	
**Marital status**				
Single	227 (56.9)	83 (55.3)	144 (57.8)	0.382
Married	164 (41.1)	66 (44)	98 (39.4)	
*Divorced*	5 (1.3)	1 (0.7)	4 (1.6)	
*Widowed*	3 (0.8)	0	3 (1.2)	
**Pre-existing health conditions**				
Diabetes	17 (4.3)	5 (3.3)	12 (4.8)	0.477
Hypertension	26 (6.5)	12 (8)	14 (5.6)	0.351
Dyslipidemia	19 (4.8)	8 (5.3)	11 (4.4)	0.677
*Cardiovascular disease*	8 (2)	3 (2)	5 (2)	0.65
*Kidney disease*	2 (0.5)	2 (1.3)	0	0.141
*Respiratory disease*	9 (2.3)	6 (4)	3 (1.2)	0.073
Other	26 (6.5)	12 (8)	14 (5.6)	0.351
None	322 (80.7)	117 (78)	205 (82.3)	0.289
**Lifestyle habits**				
**Physical activity level**				
Meets guideline	88 (22.1)	28 (18.7)	60 (24.1)	0.205
Does not meet guideline	311 (77.9)	122 (81.3)	189 (75.9)	
**Smoking**				
Yes, I smoke	137 (34.3)	50 (33.3)	87 (34.9)	0.785
No, I never smoked	222 (55.6)	83 (55.3)	139 (55.8)	
I used to smoke and I stopped	40 (10)	17 (11.3)	23 (9.2)	

*P-value: Pearson's Chi-square test and Fisher's exact test with more than 20% of expected counts <5 (respiratory, kidney and cardiovascular diseases) Characteristics written in italic had >20% of the cells with expected count <5*.

Regarding the health status, 78% of the cases and 82.3% of the controls did not report having any pre-existing health condition. The most common comorbidity was hypertension in both of the study groups (8% of the cases vs. 5.6% of the controls). However, no statistically significant difference was found among any pre-existing health condition between cases and controls. Similarly, the lifestyle habits of the participants in the two study groups did not significantly differ. Non-smokers were dominant in the two groups (55.3% of the cases vs. 55.8% of the controls). About 77.9% of the sample's participants had insufficient physical activity levels; 81.3% of the cases compared to 75.9% of the controls did not meet the guideline of 150 min of moderate physical activity per week.

### Adherence to The Mediterranean Diet

Table 2 shows the levels of the adherence to the MD and the mean ± SD of the MedDiet score among the study participants. In the total sample, the MedDiet score ranged from 16 to 44 and had a mean ± SD of 30.62 ± 5.134. About 72.4% of the participants had an average adherence to the MD (75.3% of the cases compared to 70.7% of the controls, *p* = 0.307). However, the cases had a significantly lower MedDiet score mean ± SD (29.76 ± 5.169) than the controls (31.14 ± 5.052) (*p* = 0.009).

**Table 2:** Adherence to the Mediterranean diet and MedDiet score of the participants.

**Characteristics**	**Total**	**Case group**	**Control group**	***P* value**
**AMD levels;** ***n*** **(%)**
Low	20 (5)	9 (6)	11 (4.4)	0.307^†^
Average	289 (72.4)	113 (75.3)	176 (70.7)	
High	90 (22.6)	28 (18.7)	62 (24.9)	
**MedDiet score; mean** **±** **SD**	30.62 ± 5.134	29.76 ± 5.169	31.14 ± 5.052	0.009^#^

† *P-value: Pearson's Chi-square test.*

# *P-value: Independent t-test*.

### Dietary Patterns Identification

First, the PCA method was performed, having KMO = 0.733 > 0.6 and a highly significant Bartlett test (*p* < 0.001). This method allowed the extraction of three factors that accounted for 42% of the variance in the dietary intake of the participants. The three identified factors/diets were classified as follows: (i) western diet, which showed positive loadings of fried potatoes and chips, fast food, white bread and derivatives, carbonated beverages, sweets, rice and pasta; (ii) plant-based diet that was positively correlated with vegetables, salad, olive oil, fruits, grains, legumes, and cooked vegetables; (iii) juice, fiber and fish diet, which was associated with fruit juices, brown bread and derivatives, fish and seafood. Meats and hot beverages did not show any significant loadings with the extracted factors (Supplementary Table 1).

Second, on the basis of the three extracted factors, K-means cluster analysis was carried out. Convergence was achieved after sixteen iterations, and two clusters corresponding to two dietary patterns were characterized as follows: (i) western dietary pattern, which correlated positively with the western diet but negatively with the plant-based and the juice, fiber and fish diets; (ii) prudent dietary pattern, which showed opposite correlations than those of the western dietary pattern, as shown in Table 3. The western and prudent dietary patterns included 54.4 and 45.6% of the sample's participants, respectively. When assessing the dietary patterns' distribution among the two groups of the study, the majority of the cases (56.7%) and of the controls (53%) were included in the western dietary pattern cluster, and the difference between the two groups was non-significant (*p* = 0.478).

**Table 3:** Identified clusters of dietary patterns.

**Extracted factors (diets)**	**Cluster 1**	**Cluster 2**
	***n* = 217 (54.4%)**	***n* = 182 (45.6%)**
	**Western diet**	**Prudent diet**
Factor 1 (western diet)	0.36354	−0.43345
Factor 2 (plant-based diet)	−0.3921	0.4675
Factor 3 (juice, fiber and fish diet)	−0.51441	0.61333

### Food Groups Consumption

In order to identify which food groups were responsible for the significant difference of the MedDiet score mean between the cases and controls who shared similar overall dietary patterns, the consumption of the food items included in the Mediterranean diet questionnaire was assessed and compared to the levels of consumption of the food items presented in the FFQ, in order to confirm the correlations. As shown in Table 4, compared to the controls, the participants in the case group had a significantly higher consumption of poultry (*p* = 0.014) and borderline lower intakes of olive oil (*p* = 0.079). Additionally, the statistical analysis showed a borderline significant difference in the consumption levels of red meat and products (*p* = 0.067) and alcohol (*p* = 0.076) between the cases and controls, with higher consumption levels of red meat and less moderate alcohol intake reported by the cases (Supplementary Table 2). The FFQ food items' analysis partly confirmed these results (Table 5); cases consumed less olive oil than controls (*p* = 0.004) (Supplementary Table 3).

**Table 4:** MedDiet questionnaire food items consumption.

	***P*** **value**	
**MedDiet questionnaire food items**	**Cases vs. controls**	**Females vs. males**
Non-refined cereals	0.429	0.864
Potatoes	0.149	0.14
Fruits	0.614	0.612
Vegetables	0.26	0.067^†^
Legumes	0.158	0.373
Fish	0.766	** <0.0001**
Red meat and products	0.067^†^	**0.025**
Poultry	**0.014**	**0.026**
Full fat dairy	0.943	0.613
Olive oil	0.079^†^	0.137
Alcohol	0.076^†^	**0.045**

*P value: Pearson's Chi-square test.*

† *borderline P value.*

*vs: versus.*

*Bold values indicate a significant P-value*.

**Table 5:** FFQ food items consumption.

	***P***	
**FFQ food items^**†**^**	**Cases vs. controls**	**Females vs. males**
Vegetables and salad	0.175	0.294
Fruits	0.188	0.733
Olive oil	**0.004**	0.327
Grains and legumes	0.919	0.367
Fish and sea food	0.848	0.805
Meats	0.272	**0.025**
White bread and derivatives	0.552	0.222
Brown bread and derivatives	0.724	0.665
Rice and pasta	0.077	**0.027**
Sweets	0.155	0.519
Carbonated beverages	0.528	0.17
Fruit juices	0.647	0.988
Hot beverages	0.514	0.264
Cooked vegetables	0.273	0.837
Fast food	0.327	0.653
Fried potato and chips	0.249	**0.027**

*P value: Pearson's Chi-square test.*

vs: versus.

† *Cut off for food items except olive oil in cases vs. controls: 3 times per week.*

*Bold values indicate a significant P-value*.

To further explore the factors that caused differences in the levels of food intake between the participants and consequently in the MedDiet score, gender-related food intake was assessed. Indeed, results of the Mediterranean diet questionnaire and the FFQ were greatly compatible (Tables 4, 5). Males consumed more red meat, poultry, fish, fried potatoes, chips and alcohol, but less rice and pasta than females. Moreover, the results showed a borderline significant difference in the consumption of vegetables between the two genders, with females having the higher intakes of this food group (Supplementary Tables 4, 5).

### COVID-19 Infection Characteristics and Correlates

Table 6 presents the COVID-19 characteristics in the study's sample. The majority of the cases had a moderate or a mild COVID-19 burden (49.33 and 41.33%, respectively). The most commonly reported symptoms were fatigue (75.3%), loss of smell (64.7%), and headache (60.7%). Only 2.7% of the cases were hospitalized and none of them had a severe hospitalized case.

**Table 6:** COVID-19 infection characteristics in the case group.

**Characteristics**	**Presence**	**Absence**
	***n* (%)**	***n* (%)**
**COVID-19 symptoms**		
Abdominal pain	26 (17.3)	124 (82.7)
Chest pain	45 (30)	105 (70)
Fatigue	113 (75.3)	37 (24.7)
Fever	70 (46.7)	80 (53.3)
Headache	91 (60.7)	59 (39.3)
Hoarse voice	44 (29.3)	106 (70.7)
Loss of smell	97 (64.7)	53 (35.3)
Persistent cough	51 (34)	99 (66)
Shortness of breath	43 (28.7)	107 (71.3)
Skipped meals	36 (24)	114 (76)
Sore throat	36 (24)	114 (76)
Unusual muscle pains	80 (53.3)	70 (46.7)
Diarrhea	55 (36.7)	95 (63.3)
Delirium	12 (8)	138 (92)
**Hospitalization**	4 (2.7)	146 (97.3)
**Hospitalized case severity classes**		
Severe	0	0
Non-severe	4 (100)	0

Multiple binary logistic regression tests did not correlate the sociodemographic characteristics, lifestyle habits nor the health status with the occurrence of COVID-19, except for a borderline inverse association between unemployment and the risk of COVID-19 (Supplementary Table 6). However, multiple binary logistic regression tests showed a significant inverse association between the MedDiet score and the risk of COVID-19 (OR: 1.055, CI 95%: 1.013–1.099, *p* = 0.01). Nevertheless, the western and prudent dietary patterns did not significantly influence the odds of COVID-19, as shown in Table 7.

**Table 7:** Multiple logistic regression for the association between the dietary intake and the occurrence of COVID-19.

**Characteristics**	**Odds ratio (95% CI)**	***P***
**MedDiet score**	1.055 (1.013–1.099)	**0.01**
**Dietary patterns**
Western	0.863 (0.574–1.297)	0.478
Prudent^#^	**-**	**-**

*Multiple logistic regression for the odds of being non infected by COVID-19.*

# *Reference category.*

*Bold values indicate a significant P-value*.

Concerning the correlates of the COVID-19 burden, and as presented in Table 8, multiple multinominal logistic regression tests in reference to the high COVID-19 burden showed that younger age, <50 years old, increased the odds of a moderate burden (OR: 6.455, CI 95%: 1.154–36.107, *p* = 0.034), and the absence of pre-existing health conditions increased the odds of mild (OR: 6.171, CI 95%: 1.777–21.429, *p* = 0.004) and moderate COVID-19 burden (OR: 11.52, CI 95%: 3.202–41.448, *p* < 0.0001). In particular, the absence of respiratory diseases increased the odds of mild (OR: 8.182, CI 95%: 1.222–54.767, *p* = 0.03) and moderate (OR: 19.909, CI 95%: 1.898–208.816, *p* = 0.013) COVID-19 burden compared to high burden category. Active smoking was borderline-associated with a mild COVID-19 burden (OR: 6.5, CI 95%: 0.957–44.137, *p* = 0.055). In multiple multinominal logistic regression tests adjusted for age, pre-existing health conditions and respiratory diseases, neither the MedDiet score nor the western and prudent diets affected the COVID-19 burden, as presented in Table 9.

**Table 8:** Multiple logistic regression for the association of selected study variables with the burden of COVID-19 among the case group.

	**Mild COVID-19 burden**		**Moderate COVID-19 burden**	
**Characteristics**	**Odds ratio (95% CI)**	***P* value**	**Odds ratio (95% CI)**	***P* value**
**Governorate of residence** ^**a**^		
Other	0.512 (0.159–1.654)	0.263	0.574 (0.182–1.814)	0.345
Bekaa^#^	**-**	**-**	**-**	**-**
**Gender**		
Female	0.556 (0.167–1.847)	0.338	2.87 (0.818–10.078)	0.1
Male^#^	**-**	**-**	**-**	**-**
**Age group in years**		
21–49	2.545 (0.552–11.746)	0.231	6.455 (1.154–36.107)	**0.034**
50–64^#^	**-**	**-**	**-**	**-**
**Educational level**		
Pre-university level^b^	1.294 (0.253–6.622)	0.757	0.831 (0.159–4.331)	0.826
University and/or higher education^#^	**-**	**-**	**-**	**-**
**Employment status**		
Not working^c^	0.426 (0.127–1.424)	0.166	0.722 (0.226–2.306)	0.583
Working^#^	**-**	**-**	**-**	**-**
**Marital status**		
Single^d^	1.583 (0.494–5.079)	0.44	1.114 (0.355–3.494)	0.853
Married^#^	**-**	**-**	**-**	**-**
**Pre-existing health conditions**		
Absence of diseases	6.171 (1.777–21.429)	**0.004**	11.52 (3.202–41.448)	** <0.0001**
Presence of diseases^#^	**-**	**-**	**-**	**-**
Absence of diabetes	1.513 (0.146–15.728)	0.729	5.615 (0.33–95.528)	0.233
Absence of hypertension	3.109 (0.647–14.947)	0.157	4.773 (0.939–24.27)	0.06
Absence of dyslipidemia	3.278 (0.493–21.779)	0.219	3.944 (0.595–26.133)	0.155
Absence of cardiovascular diseases	10.167 (0.852–121.278)	0.067	N/A	N/A
Absence of kidney diseases	4.692 (0.275–79.971)	0.285	N/A	N/A
Abence of respiratory diseases	8.182 (1.222–54.767)	**0.03**	19.909 (1.898–208.816)	**0.013**
Absence of other diseases	3.109 (0.647–14.947)	0.157	4.773 (0.939–24.27)	0.06
**Smoking**		
Yes, I smoke	6.5 (0.957–44.137)	0.055	2.1 (0.358–12.312)	0.411
No, I never smoked	3 (0.556–16.186)	0.201	1.612 (0.362–7.189)	0.531
I used to smoke and I stopped^#^	**-**	**-**	**-**	**-**
**Physical activity level**		
Meets the guideline	1.915 (0.384–9.540)	0.428	1.048 (0.206–5.337)	0.955
Does not meet the guideline ^#^	**-**	**-**	**-**	**-**

*Multiple logistic regression in reference to the high COVID-19 burden category.*

a *Other included South Lebanon, Beirut, Akkar, Mount Lebanon, Nabatieh, North Lebanon and Baalbek – Hermel.*

b *Pre-university level included elementary, middle or high school.*

c *Not working included student, not working or retired*.

d *Single included single, divorced or widowed*.

N/A, not applicable.

# *Reference category.*

*Bold values indicate a significant P-value*.

**Table 9:** Multiple adjusted logistic regression for the association between the dietary intake and the burden of COVID-19.

	**Mild COVID-19 burden**		**Moderate COVID-19 burden**	
**Characteristics**	**Odds ratio (95% CI)**	***P*value**	**Odds ratio (95% CI)**	***P*value**
**MedDiet score**	0.946 (0.832–1.077)	0.401	0.992 (0.873–1.129)	0.908
**Dietary patterns**
Western	0.384 (0.097–1.528)	0.175	0.553 (0.138–2.219)	0.403
Prudent^#^	**-**	**-**	**-**	**-**

*Multiple logistic regression adjusted for age, pre-existing health conditions and respiratory diseases in reference to the high COVID-19 burden category.*

# *Reference category.*

## Discussion

This research study aimed at exploring the association between the dietary intake and the occurrence and burden of COVID-19. Among our sample of Lebanese adults, the biggest proportion of the participants in both of the study groups had an average adherence to the MD. Only 22.6% of the total sample had a high level of adherence to this diet, which gives a recall to two studies done in Lebanon that found that 24 and 18.8% of the adults included in their samples had a high adherence to the MD (21, 22).

Data-driven methods allowed the identification of three diets, namely the western, plant-based and juice, fiber, and fish diets. Further analysis of the dietary intake resulted in grouping the participants into two clusters of dietary patterns labeled as western and prudent. In the two groups of the study, we found a dominance of the western dietary pattern. Actually, western-type dietary patterns and healthy dietary patterns close to the prudent diet found in our study have been previously identified among the Lebanese population in other research (17, 23–26). Nonetheless, the vast popularity of the western diet in our sample might be explained by the nutrition transition in the Lebanese setting, which was characterized by a shift toward the western-type foods and a decrease in the consumption of nutritionally dense foods (10).

Interestingly, although they shared overall similar dietary patterns, participants in the case group had a lower MedDiet score mean than the controls. This difference was found to be caused by a higher consumption of poultry and a trend toward lower intake of olive oil and increased red meat and alcohol consumption among the cases. Indeed, a high consumption of animal protein has been correlated with pro-inflammatory effects (27). In a recent study, meat consumers had a less varied and less balanced dietary intake than low-meat consumers, which might influence the general nutritional status of the individuals and consequently affect the immune response (28). Moreover, olive oil has been associated with anti-inflammatory and antioxidant effects (5). More specifically, it was suggested that the consumption of olive oil could improve vitamin D status, potentially decreasing the risk of COVID-19, knowing that a vitamin D deficiency and a pro-inflammatory status are among the COVID-19 risk factors (29–32). Furthermore, non-moderate alcohol intakes might affect the lungs' immunity (33). Our findings also showed that gender influenced the participant's food choices. Males consumed more red meat, poultry, fish, alcohol, fried potatoes and chips, while females had significantly higher intakes of rice and pasta, and a borderline higher consumption of vegetables. In fact, the male preference for high-fat foods and the female inclination toward carbohydrates-rich foods have previously been reported (34).

Regarding the COVID-19 infection, the majority of the cases had a moderate or mild infection burden, which is in line with the findings of Lebanese COVID-19 cohort where 86.3% of the participants had moderate COVID-19 cases (35). When assessing the correlates of the infection occurrence, gender, age, physical activity, smoking, pre-existing health conditions and other sociodemographic characteristics did not influence the odds of COVID-19, which can be rationalized by the followings. First, gender-related differences in regard to the risk of the infection are still inconclusive, and it has been stated that both genders have a similar risk of presenting the infection (36). Second, the 20–29 years old age group in Lebanon was reported to present a high rate of infection, which indicates that COVID-19 can affect several age groups and not only older adults (11). Third, the vast dominance of insufficient physical activity levels in both of our study's groups might have prevented a significant association with the occurrence of COVID-19. Fourth, smoking rates among COVID-19 cases in a study in France were lower than those of the general population, which gives a recall to the absence of smoking's influence on the occurrence of COVID-19 (37). Fifth, a study in Korea did not correlate several comorbidities with the increased risk of COVID-19 due to a possibility of a limited social interaction of these individuals (38). It should be noted that our results found a borderline association between unemployment and a decreased risk of COVID-19, possibly linked to the less frequent social contact of unemployed individuals.

The increased MedDiet score values were associated with a lower risk of COVID-19 occurrence. This result is compatible with the findings of a recent ecological study that showed an inverse correlation between the adherence to the MD and COVID-19 cases in selected European countries (9). This association can be attributed to the anti-inflammatory profile of the MD and its optimal nutritional quality. This diet provides several nutrients that are needed for immunocompetence such as mono-unsaturated fatty acids, omega-3 fatty acids, flavonoids, vitamins C, E, β-carotene, selenium, folate, fibers, therefore conferring immune support in the context of COVID-19 (5). However, this association between the MD and the risk of COVID-19 could not be fully confirmed due to the followings. First, the majority of the cases and controls did not properly adhere to the MD and had a western-like dietary pattern. Second, the difference between the means of the MedDiet score between the two groups of the study was relatively small. Therefore, the preventive role of the MD in the context of COVID-19 could not be validated solely based on the study's results.

Regarding the western and prudent dietary patterns identified in our study, they did not influence the occurrence of COVID-19, which might be explained by some limitations of the dietary patterns' identification methods. In fact, the dietary intake is a combination of different food items complexly interacting with each other. Thus, limiting the characterization of the overall dietary intake to a simple qualification of one pattern “leaves sufficient room for other patterns to prevail” (20). Moreover, the different interactions between the consumed amounts of foods that either have pro- or anti-inflammatory effects might maintain a balanced inflammatory state and mask the individual effect of specific foods (39).

Our findings showed an association between advanced age and pre-existing health conditions, specifically respiratory diseases, with the higher COVID-19 burden. Indeed, older age and the presence of comorbidities have been recognized as major risk factors of the infection's severity (40, 41). Other individual characteristics did not influence the burden of COVID-19 in our study. In fact, gender-related differences in the severity of COVID-19 are not confirmed due to complexity of the physiological differences and similarities between males and females (42). Surprisingly, physical activity levels did not impact the infection's burden, in opposite to the findings of a recent study that showed an inverse association between physical activity practice and COVID-19 severity (43). Although the majority of evidence has associated smoking with a negative infection prognosis, some studies have reported a non-significant influence of smoking on the severity of COVID-19 (37, 44). This is postulated to be related to the angiotensin converting enzyme 2 (ACE2) possible regulatory effect of nicotine, as well to its role in decreasing tumor necrosis factor (TNF) expression in the airways and in serving as an agonist of the cholinergic anti-inflammatory pathway that influences the immune and inflammatory responses (45).

Our study did not correlate the dietary intake with any significant changes of the infection's burden. Although incompatible with the findings of the previously referred-to study in Europe, which found that the adherence to the MD was correlated with lower COVID-19 death rates, our results might be explained by the followings (9). First, the use of nutritional supplements throughout the infection has increased and might have influenced the infection's outcomes (46). Second, other determining factors such as genetics, medications and obesity were not assessed in our study and might have impacted the association between diets and the COVID-19 burden (47, 48). Third, the dietary patterns might not have reflected the individual differences in the food consumption, but rather gave a general overview of the population's dietary intake (20).

Our study presents several strengths. The simultaneous use of an *a priori* and two *a posteriori* methods allowed a complementary evaluation of the dietary intake. Additionally, the assessment of the food groups' consumption by the Mediterranean diet questionnaire and the FFQ allowed confirmation of the observed associations.

As for the limitations, the observational type of our study could not lead to causative associations. Although the sample was statistically representative of the population, the limited participation of COVID-19 cases has prevented more powerful statistical investigations. The questionnaire was self-administrated, which could have led to biased responses. Additionally, there was a possibility of a recall and estimation bias because the participants might have been affected by the COVID-19 repercussions or might have been infected a long time before taking part in this study, which could have limited their motivation or their accurate estimation of their dietary intake. Moreover, the dietary patterns are an estimation of the overall dietary intake but could not expose inter-individual dietary variations that might mitigate the immune response to COVID-19. Last but not least, other aspects that affect the occurrence and the outcomes of the infection were not assessed, such as the exposure to infected carriers, the medications prescribed and other previously stated factors.

In conclusion, our findings showed that an increased intake of poultry and a trend toward decreased consumption of olive oil and increased read meat and alcohol intake were observed among the COVID-19 cases compared to healthy controls. Therefore, healthy food choices are needed within the varied dietary patterns as they can lead to differences in the nutritional profile and consequently influence the risk of COVID-19. However, the limited differences of the MedDiet score between the two groups of the study, as well as the adoption of the western dietary pattern by both cases and controls did not allow the affirmation of the inverse association between the MD and COVID-19 occurrence. This study could serve as a basis for larger-scale studies that might include a bigger proportion of cases and investigate several characteristics that might influence the infection's occurrence and outcomes. In particular, further research that explore the possible association between the MD and the occurrence of COVID-19 are needed to confirm our findings. This study's results could be used as a tool for public health management and for raising awareness about the importance of optimal nutrition in the COVID-19 pandemic.

## Resource Identification Initiative

To take part in the Resource Identification Initiative, please use the corresponding catalog number and RRID in your current manuscript. For more information about the project and for steps on how to search for an RRID, please click here.

## Data Availability Statement

The raw data supporting the conclusions of this article will be made available by the authors, without undue reservation.

## Author Contributions

CEK contributed to the conceptualization, investigation, and design of the study together with SGJ. CEK also organized the database, performed the statistical analysis, and wrote the original draft of the manuscript. SGJ supervised, reviewed, and validated the manuscript. CEK and SGJ approved the final version for submission. All authors contributed to the article and approved the submitted version.

## Conflict of Interest

The authors declare that the research was conducted in the absence of any commercial or financial relationships that could be construed as a potential conflict of interest.

## Publisher's Note

All claims expressed in this article are solely those of the authors and do not necessarily represent those of their affiliated organizations, or those of the publisher, the editors and the reviewers. Any product that may be evaluated in this article, or claim that may be made by its manufacturer, is not guaranteed or endorsed by the publisher.
