# Supplementary material for: Inverse Association Between the Mediterranean Diet and COVID-19 Risk in Lebanon: A Case-Control Study
Source: Front Nutr. 2021 Jul 30;8:707359. doi: 10.3389/fnut.2021.707359 (PMC8363114; doi:10.3389/fnut.2021.707359)
Supplement: Supplementary file 2 [file Table_1.docx]

**Supplementary Table 1.** Pattern loading of the three factors solutions after varimax rotation

|  | **Extracted factors** | | |  |
| --- | --- | --- | --- | --- |
| **Food items** | **1** | **2** | **3** |  |
| Fried potatoes and chips | 0.719 |  |  |  |
| Fast food | 0.715 |  |  |  |
| White bread and derivatives | 0.7 |  |  |  |
| Carbonated beverages | 0.553 |  | 0.414 |  |
| Sweets | 0.507 |  |  |  |
| Rice and pasta | 0.502 |  |  |  |
| Vegetables and salad |  | 0.77 |  |  |
| Olive oil |  | 0.694 |  |  |
| Fruits |  | 0.645 |  |  |
| Grains and legumes |  | 0.531 |  |  |
| Cooked vegetables |  | 0.525 |  |  |
| Fruit juices |  |  | 0.631 |  |
| Brown bread and derivatives |  |  | 0.576 |  |
| Fish and seafood |  |  | 0.509 |  |
| *Total variance explained by the three factors is 42%. Absolute values <0.4 were excluded from the table for simplicity. "Meats" and "hot beverages" were excluded as they did not have significant loadings for any of the extracted factors.* | | | | |
