# Supplementary material for: Inverse Association Between the Mediterranean Diet and COVID-19 Risk in Lebanon: A Case-Control Study
Source: Front Nutr. 2021 Jul 30;8:707359. doi: 10.3389/fnut.2021.707359 (PMC8363114; doi:10.3389/fnut.2021.707359)
Supplement: Supplementary file 3 [file Table_2.docx]

**Supplementary Table 2.** Levels of consumption of the MedDiet questionnaire food items between cases and controls

| **MedDiet questionnaire food items** | **Case group** | **Control group** | ***P* value** |
| --- | --- | --- | --- |
|  | **n (%)** | **n (%)** |  |
| **Red meat and products** |  |  |  |
| Never | 3 (2) | 7 (2.8) | 0.067^†^ |
| 1- 4 servings per month | 26 (17.3) | 55 (22.1) |  |
| 5 - 8 servings per month | 33 (22) | 64 (25.7) |  |
| 9 - 12 servings per month | 46 (30.7) | 59 (23.7) |  |
| 13 - 18 servings per month | 23 (15.3) | 50 (20.1) |  |
| More than 18 servings per month | 19 (12.7) | 14 (5.6) |  |
| **Poultry** |  |  |  |
| Never | 1 (0.7) | 5 (2) | **0.014** |
| 1 - 4 servings per month | 13 (8.7) | 36 (14.5) |  |
| 5 - 8 servings per month | 34 (22.7) | 67 (26.9) |  |
| 9- 12 servings per month | 51 (34) | 73 (29.3) |  |
| 13 - 18 servings per month | 25 (16.7) | 50 (20.1) |  |
| More than 18 servings per month | 26 (17.3) | 18 (7.2) |  |
| **Olive oil** |  |  |  |
| Never | 4 (2.7) | 11 (4.4) | 0.079^†^ |
| Rare | 15 (10) | 30 (12) |  |
| Less than 1 serving per week | 11 (7.3) | 20 (8) |  |
| 1 - 3 servings per week | 47 (31.3) | 73 (29.3) |  |
| 3 - 5 servings per week | 34 (22.7) | 30 (12) |  |
| Daily | 39 (26) | 85 (34.1) |  |
| **Alcohol** |  |  |  |
| Less than 300 ml per day | 100 (66.7) | 184 (73.9) | 0.076^†^ |
| 300 ml per day | 7 (4.7) | 16 (6.4) |  |
| 400 ml per day | 3 (2) | 9 (3.6) |  |
| 500 ml per day | 3 (2) | 6 (2.4) |  |
| 600 ml per day | 0 | 0 |  |
| More than 700 or 0 ml per day | 37 (24.7) | 34 (13.7) |  |
| *P value: Pearson's Chi-square test ^†^ borderline P value*  *Bold value indicates a significant P-value* | | | |
