# Supplementary material for: Inverse Association Between the Mediterranean Diet and COVID-19 Risk in Lebanon: A Case-Control Study
Source: Front Nutr. 2021 Jul 30;8:707359. doi: 10.3389/fnut.2021.707359 (PMC8363114; doi:10.3389/fnut.2021.707359)
Supplement: Supplementary file 4 [file Table_3.docx]

**Supplementary Table 3.** Levels of consumption of the FFQ food item between cases and controls

| **Food item** | **Case group** | **Control group** | ***P* value** |
| --- | --- | --- | --- |
|  | **n (%)** | **n (%)** |  |
| **Olive oil** |  |  |  |
| Never^†^ | 2 (1.3) | 4 (1.6) | **0.004** |
| 1 or 2 times per week | 45 (30) | 78 (31.3) |  |
| 3 to 6 times per week | 75 (50) | 84 (33.7) |  |
| Daily | 28 (18.7) | 83 (33.3) |  |
| *P value: Pearson's Chi-square test ^†^ expected count less than 5.*  *Bold value indicates a significant P-value* | | | |
