# Supplementary material for: Inverse Association Between the Mediterranean Diet and COVID-19 Risk in Lebanon: A Case-Control Study
Source: Front Nutr. 2021 Jul 30;8:707359. doi: 10.3389/fnut.2021.707359 (PMC8363114; doi:10.3389/fnut.2021.707359)
Supplement: Supplementary file 5 [file Table_4.docx]

**Supplementary Table 4.** Levels of consumption of the MedDiet questionnaire food items between females and males

|  | **Gender** | |  |
| --- | --- | --- | --- |
| **MedDiet questionnaire food items** | **Female** | **Male** | ***P* value** |
|  | **n (%)** | **n (%)** |  |
| **Vegetables** |  |  |  |
| Never | 1 (0.4) | 3 (2.5) | 0.067^†^ |
| 1- 4 servings per month | 16 (5.7) | 13 (10.8) |  |
| 5 - 8 servings per month | 36 (12.9) | 16 (13.3) |  |
| 9 - 12 servings per month | 46 (16.5) | 25 (20.8) |  |
| 13 - 18 servings per month | 67 (24) | 27 (22.5) |  |
| More than 18 servings per month | 113 (40.5) | 36 (30) |  |
| **Fish** |  |  |  |
| Never | 27 (9.7) | 12 (10) | **<0.0001** |
| 1 - 4 servings per month | 179 (64.2) | 57 (47.5) |  |
| 5 - 8 servings per month | 52 (18.6) | 24 (20) |  |
| 9- 12 servings per month | 14 (5) | 19 (15.8) |  |
| 13 - 18 servings per month^#^ | 4 (1.4) | 8 (6.7) |  |
| More than 18 servings per month^#^ | 3 (1.1) | 0 |  |
| **Red meat and products** |  |  |  |
| Never | 7 (2.5) | 3 (2.5) | **0.025** |
| 1 - 4 servings per month | 68 (24.4) | 13 (10.8) |  |
| 5 - 8 servings per month | 65 (23.3) | 32 (26.7) |  |
| 9- 12 servings per month | 75 (26.9) | 30 (25) |  |
| 13 - 18 servings per month | 45 (16.1) | 28 (23.3) |  |
| More than 18 servings per month | 19 (6.8) | 14 (11.7) |  |
| **Poultry** |  |  |  |
| Never | 4 (1.4) | 2 (1.7) | **0.026** |
| 1 - 4 servings per month | 38 (13.6) | 11 (9.2) |  |
| 5 - 8 servings per month | 73 (26.2) | 28 (23.3) |  |
| 9- 12 servings per month | 87 (31.2) | 37 (30.8) |  |
| 13 - 18 servings per month | 56 (20.1) | 19 (15.8) |  |
| More than 18 servings per month | 21 (7.5) | 23 (19.2) |  |
| **Alcohol** |  |  |  |
| Less than 300 ml per day | 204 (73.1) | 80 (66.7) | **0.045** |
| 300 ml per day | 11 (3.9) | 12 (10) |  |
| 400 ml per day | 6 (2.2) | 6 (5) |  |
| 500 ml per day | 5 (1.8) | 4 (3.3) |  |
| 600 ml per day | 0 | 0 |  |
| More than 700 or 0 ml per day | 53 (19) | 18 (15) |  |
| *P value: Pearson's Chi-square test † borderline P value ^#^ expected count less than 5*  *Bold values indicate a significant P-value* | | | |
