# Supplementary material for: Inverse Association Between the Mediterranean Diet and COVID-19 Risk in Lebanon: A Case-Control Study
Source: Front Nutr. 2021 Jul 30;8:707359. doi: 10.3389/fnut.2021.707359 (PMC8363114; doi:10.3389/fnut.2021.707359)
Supplement: Supplementary file 6 [file Table_5.docx]

**Supplementary Table 5.** Levels of consumption of the FFQ food items between females and males

| **FFQ food items** | **Females** | **Males** | ***P* value** |
| --- | --- | --- | --- |
|  | **n (%)** | **n (%)** |  |
| **Meats** |  |  |  |
| Less than 3 times per week | 141 (50.5) | 46 (38.3) | **0.025** |
| More than 3 times per week | 138 (49.5) | 74 (61.7) |  |
| **Rice and pasta** |  |  |  |
| Less than 3 times per week | 160 (57.3) | 83 (69.2) | **0.027** |
| More than 3 times per week | 119 (42.7) | 37 (30.8) |  |
| **Fried potato and chips** |  |  |  |
| Less than 3 times per week | 203 (72.8) | 74 (61.7) | **0.027** |
| More than 3 times per week | 76 (27.2) | 46 (38.3) |  |
| *P value: Pearson's Chi-square test*  *Bold values indicate a significant P-value* | | | |
