# Supplementary material for: Inverse Association Between the Mediterranean Diet and COVID-19 Risk in Lebanon: A Case-Control Study
Source: Front Nutr. 2021 Jul 30;8:707359. doi: 10.3389/fnut.2021.707359 (PMC8363114; doi:10.3389/fnut.2021.707359)
Supplement: Supplementary file 7 [file Table_6.docx]

**Supplementary Table 6.** Multiple logistic regression for the association of selected study variables with the occurrence of COVID-19 among the study participants

| **Characteristics** | **Odds ratio (95% CI)** | ***P* value** |
| --- | --- | --- |
| **Governorate of residence ^a^** | | |
| Bekaa | 0.834 (0.55 - 1.266) | 0.395 |
| Other^#^ | ⁃ | ⁃ |
| **Gender** | | |
| Female | 1.157 (0.746 - 1.795) | 0.515 |
| Male^#^ | ⁃ | ⁃ |
| **Age group in years** | | |
| 21 - 49 | 0.612 (0.304 - 1.231) | 0.168 |
| 50 - 64^#^ | **⁃** | **⁃** |
| **Educational level** | | |
| Pre-university level ^b^ | 0.678 (0.369 - 1.246) | 0.211 |
| University and/or higher education^#^ | **⁃** | **⁃** |
| **Employment status** | | |
| Not working ^c^ | 1.471 (0.959 - 2.257) | 0.077 |
| Working^#^ | **⁃** | **⁃** |
| **Marital status** | | |
| Single ^d^ | 1.211 (0.803 - 1.825) | 0.362 |
| Married^#^ | **⁃** | **⁃** |
| **Pre-existing health conditions** | | |
| Absence | 1.314 (0.793 - 2.178) | 0.289 |
| Presence^#^ | ⁃ | ⁃ |
| **Smoking** | | |
| Yes, I smoke | 1.286 (0.628 - 2.634) | 0.492 |
| No, I never smoked | 1.238 (0.625 - 2.451) | 0.541 |
| I used to smoke and I stopped ^#^ | **⁃** | **⁃** |
| **Physical activity level** | | |
| Meets the guideline | 1.383 (0.836 - 2.287) | 0.206 |
| Does not meet the guideline ^#^ | ⁃ | ⁃ |
| *Multiple logistic regression in reference to the non-COVID-19 group ^a^ Other included South Lebanon, Beirut, Akkar, Mount Lebanon, Nabatieh, North Lebanon and Baalbek - Hermel ^b^ Pre-university level included elementary, middle or high school ^c^ Not working included student, not working or retired ^d^ Single included single, divorced or widowed ^#^ Reference category* | | |
